# Socio-Educational Ambivalence in Intercultural Contexts: A Comparative Analysis of Teachers and Students in Schools in Mapuche Contexts

**DOI:** 10.3390/bs16061003

**Published:** 2026-06-16

**Authors:** Daniel Quilaqueo, Enrique Riquelme-Mella, Flavio Muñoz-Troncoso, Héctor Torres, Gloria Mora-Guerrero

**Affiliations:** 1 Department of Diversity and Intercultural Education, Faculty of Education, Universidad Católica de Temuco, Temuco 4810296, Chile; dquilaq@uct.cl; 2 Department of Education and Innovation, Faculty of Education, Universidad Católica de Temuco, Temuco 4810296, Chile; 3 Faculty of Social Sciences and Arts, Universidad Mayor, Temuco 4801043, Chile; 4 International Observatory on School Climate and Violence Prevention (IOSCVP), 41004 Sevilla, Spain; 5 Department of Educational Sciences, Faculty of Education and Humanities, Campus Fernando May, Universidad del Bio-Bio, Chillán 3800708, Chile; htorres@ubiobio.cl; 6 School of Psychology, Faculty of Humanities, Universidad de Santiago de Chile, Santiago 9170022, Chile; gloria.mora@usach.cl

**Keywords:** intercultural education, socio-educational ambivalence, Mapuche, teachers, students, educational system

## Abstract

Intercultural education in Mapuche contexts is shaped by persistent tensions between dominant school knowledge and Indigenous educational practices. However, there is limited comparative empirical evidence on how these tensions are distributed across educational actors. This study aimed to compare socio-educational and cultural ambivalence between students and teachers across multiple dimensions. A cross-sectional quantitative design was conducted with 546 participants (284 students and 262 teachers) from primary and secondary schools in southern Chile. Ambivalence was assessed using the Socio-Educational and Cultural Ambivalence Scale (EASC). A two-step cluster analysis identified ambivalence profiles, followed by a 2 × 2 factorial MANOVA (role × ethnicity). Results revealed three distinct ambivalence profiles (low, medium, high), with significant differences across all dimensions (*p* < 0.001). Multivariate analyses showed significant effects of role (Pillai’s trace = 0.230, F (6, 537) = 26.67, *p* < 0.001, η^2^p = 0.230) and ethnicity (Pillai’s trace = 0.108, F (6, 537) = 10.86, *p* < 0.001, η^2^p = 0.108), with no significant multivariate interaction (*p* = 0.104). Teachers reported higher levels of ambivalence than students in five of six dimensions, while Mapuche participants scored higher than non-Mapuche participants across most dimensions. These findings indicate that ambivalence is a structural condition of the educational system, unevenly distributed according to actors’ positions and intensified in roles involving pedagogical mediation. Implications point to the need for structural transformations in intercultural education, particularly in teacher education.

## 1. Introduction

Intercultural education in Chile is a field marked by persistent epistemological tensions, especially in territories with a historical presence of indigenous peoples, such as the Mapuche people in the Araucanía Region. Despite the implementation of policies such as the Intercultural Bilingual Education Program (PEIB) since 1996, various studies agree that these initiatives have had a limited impact on the transformation of pedagogical practices, curricular content, and power relations in the school system (Quilaqueo et al., 2016; Quilaqueo & Torres, 2013). In this context, the school continues to operate predominantly under a monocultural rationality with a Eurocentric matrix, which reproduces forms of coloniality of knowledge. In this scenario, the school experience in Mapuche contexts cannot be understood only from indicators of academic access or achievement, but requires considering the cultural, cognitive and affective tensions that emerge from the coexistence of different knowledge systems: the dominant school knowledge and the Mapuche educational knowledge present in the family and the community. These tensions have been conceptualized as socio-educational and cultural ambivalence, understood as the coexistence of potentially contradictory value, epistemic, and affective orientations in educational actors.

From a classical sociological perspective, ambivalence constitutes a structural disposition derived from the coexistence of incompatible normative expectations (Simmel, 1971; Merton, 1968). In the intercultural educational field, this phenomenon is expressed as a tension between differentiated educational rationalities, where subjects must simultaneously adhere to dominant school norms and sustain their own cultural references (Quilaqueo et al., 2016; Quilaqueo & Torres, 2022). Empirical evidence suggests that this ambivalence is not restricted to students, but rather crosses different actors in the school system. Teachers, for example, face tensions when trying to incorporate indigenous knowledge into a curricular framework that does not fully recognize its legitimacy, while families experience contradictions between school expectations and their own educational conceptions. However, despite its theoretical relevance, there is a paucity of comparative quantitative studies that analyze how ambivalence is distributed among educational actors in intercultural contexts. Therefore, the present study aims to compare the socio-educational and cultural ambivalence between students and teachers in Mapuche school contexts, considering its expression in different dimensions of the construct. Specifically, it seeks to identify differences in the levels of ambivalence according to role and ethnicity, contributing to a more precise understanding of the intercultural dynamics that shape the school experience in indigenous territories.

### 1.1. Intercultural Education in Mapuche Territory: Structural Tensions of the School System

Intercultural education in Chile has been defined as a process aimed at promoting respectful pedagogical relations between the school and indigenous peoples. However, the accumulated evidence shows that its implementation has had a limited scope in terms of structural transformation of the education system (Sotomayor et al., 2014; Luna et al., 2018; Becerra-Lubies et al., 2024). Various studies have pointed out that the intercultural approach promoted by the State in Latin America has operated fundamentally as a complement to the curriculum, without questioning the epistemological bases that organize modern schooling (Walsh, 2010; Tubino & Sinnigen, 2013). In this sense, institutionalized interculturality has not implied a reconfiguration of the education system, but rather its superficial adaptation to contexts of cultural diversity (Estermann, 2020). The dominant school model continues to be structured around a monocultural rationality, which privileges abstract, decontextualized, and universalist forms of knowledge, reproducing what various authors have characterized as persistent forms of coloniality of knowledge (Santos, 2009; Fornet-Betancourt, 2018). This rationality not only defines curricular content, but also the criteria for validating knowledge, the forms of teaching, and evaluation mechanisms (Bertely, 2008; Olivé, 2009).

In the case of the Mapuche people, this situation acquires a particular historical density. The school has operated as a device for cultural assimilation since its installation in the territory, first through missionary projects and later through the state public system. The incorporation of Intercultural Bilingual Education (IBE) since the 1990s has not reversed this logic, as its implementation has been fragmented, dependent on local initiatives, and sustained by peripheral figures such as the Traditional Educators (ELCI) (Quilaqueo et al., 2016).

As a result, the education system configures a scenario in which heterogeneous forms of knowledge and educational practices coexist—without effective articulation. This coexistence is not neutral, but is organized hierarchically, privileging the dominant school knowledge and subordinating Mapuche educational knowledge. Consequently, intercultural education does not eliminate tensions, but rather reconfigures them within the school space, producing structural conditions of conflict that run through the educational experience.

### 1.2. Double Educational Rationality

The notion of double educational rationality allows us to understand more precisely the nature of these tensions. This approach states that in the Mapuche school context two systems of knowledge coexist with differentiated epistemological foundations: On the one hand, Western educational rationality, characterized by abstraction, disciplinary sequencing, and universality; and on the other, Mapuche educational rationality, based on relationality, territoriality, orality, and community experience (Quilaqueo et al., 2017). This coexistence does not imply complementarity, but rather configures a structural epistemic conflict. While the school system requires the internalization of decontextualized content and adherence to universal criteria of validity, Mapuche knowledge is organized around situated practices, linked to the territory and to forms of community life such as the küme mongen. The divergence between these rationalities is not limited to content, but crosses the ways of learning, teaching and understanding the world.

In this framework, educational actors do not operate in a homogeneous space, but in a stressed field in which they must simultaneously respond to partially incompatible logics. This structural condition forces the development of adjustment strategies that, far from resolving the conflict, tend to reproduce it in daily practice. In this way, double rationality is not only a feature of the educational system, but also the foundation of the tensions that are expressed in the experience of the subjects, such as socio-educational ambivalence. It is in this scenario that socio-educational and cultural ambivalence is configured as a central analytical category. From classical sociology, ambivalence has been understood as a structural disposition derived from the coexistence of contradictory normative expectations (Simmel, 1971; Merton & Barber, 1963). In modern societies, this condition is not exceptional, but constitutive, insofar as individuals must articulate divergent orientations in their social action (Tabboni, 2008).

Transferred to the intercultural educational field, ambivalence can be understood as the subjective and practical expression of the epistemic conflict that emerges from the double educational rationality. In this sense, it is not an individual contradiction or a cognitive deficit, but a structural condition of the education system, in which actors must operate simultaneously from different frames of reference (Quilaqueo & Torres, 2013).

This ambivalence manifests itself at different levels. At the cognitive level, it implies the coexistence of divergent interpretative schemes; at the affective level, it is expressed in identity and value tensions; and at a practical level, it translates into pedagogical decisions, forms of participation and modes of relationship in the classroom. In this way, ambivalence not only reflects the existence of two rationalities, but also the structural difficulties for their articulation in the school context. A fundamental element in understanding socio-educational ambivalence is its differential distribution according to the position of the actors in the school system. In line with the sociological theory of roles, normative expectations and institutional demands are not distributed homogeneously, but depend on the location of subjects in the social structure (Merton, 1968).

In the case of Mapuche students, ambivalence is configured at the intersection between processes of school socialization and community socialization. These actors must adapt to a system that tends to devalue or make invisible their family knowledge, generating tensions between cultural belonging and school success. This condition can translate into processes of identity negotiation, displacement of cultural practices or forms of symbolic resistance (Quilaqueo et al., 2017).

In the case of teachers, ambivalence takes on a different and, in many cases, more complex configuration. From their professional role, teachers are in a position of mediation between knowledge systems, facing the need to incorporate Mapuche knowledge into a curricular framework that does not fully recognize them as valid. This situation is aggravated by the limited intercultural training in initial teacher training, which restricts the tools available to manage these tensions (Gay, 2010; Sleeter, 2011). Consequently, teachers’ ambivalence reflects cultural tensions, and institutional and professional contradictions.

For their part, Mapuche families experience ambivalence in the relationship between school and community educational practices, facing the need to support their children’s school trajectory without renouncing their own forms of knowledge transmission (Quilaqueo et al., 2022). However, unlike students and teachers, their position in the education system is indirect, which configures a different relationship with structural tensions.

Based on the above, the comparative analysis between students and teachers is central to understanding how socio-educational and cultural ambivalence is structured within intercultural school contexts. Rather than identifying merely descriptive group differences, this comparison makes it possible to examine how structural tensions associated with intercultural education are distributed according to actors’ institutional positions. In particular, it allows us to assess whether ambivalence is more strongly expressed among teachers, who must mediate between curricular demands and Indigenous knowledge systems, or among students, who experience the tension between cultural belonging and school demands in their educational trajectories.

At the same time, considering ethnicity is necessary to examine whether these tensions are intensified among Mapuche participants, especially in dimensions related to cultural identity, epistemic recognition, and participation in intercultural practices. Thus, socio-educational ambivalence is understood in this study not only as an individual or group experience, but also as an analytical indicator of the structural tensions of intercultural schooling. Accordingly, the general objective of this study is to compare socio-educational and cultural ambivalence between teachers and students in Mapuche school contexts, considering the role of ethnicity in the expression of this construct. Specifically, the study aims to:

(a) Compare levels of socio-educational and cultural ambivalence according to educational role (teachers vs. students) and ethnicity (Mapuche vs. non-Mapuche);
(b) Identify the dimensions of ambivalence in which these differences are expressed;
(c) Examine whether the relationship between educational role and ambivalence varies according to ethnicity;
(d) Explore ambivalence profiles as complementary configurations of intercultural educational experience.

The study considers six dimensions of the Socio-Educational and Cultural Ambivalence Scale (EASC): (F1) Integration of Contextual and Cultural Knowledge; (F2) School–Community Linkage and Knowledge Mobilization; (F3) Equal Treatment and Recognition of Cultural Diversity; (F4) Cultural Identity and Resistance to Homogenization; (F5) Intercultural Education and Deepening of Traditional Knowledge; and (F6) Participation in Cultural Ceremonies and Hybridization Spaces.

Based on the theoretical framework of structural ambivalence and double educational rationality, three hypotheses are proposed. First, teachers are expected to report higher levels of socio-educational and cultural ambivalence than students, given their institutional role as mediators between school knowledge and Mapuche educational knowledge. Second, Mapuche participants are expected to show higher scores in dimensions associated with cultural identity, epistemic recognition, and intercultural participation. Third, role and ethnicity are expected to interact in specific dimensions of ambivalence, reflecting differentiated forms of exposure to intercultural tensions.

## 2. Materials and Methods

### 2.1. Participants

A total of 546 people participated, of which 52.0% were students (*n* = 284) and 48.0% were teachers (*n* = 262), from primary and secondary schools located in the regions of La Araucanía (85.1%) and Los Lagos (14.9%). The age of the teachers ranged from 23 to 72 years (M = 42.32; SD = 10.37), while that of students was between 10 and 18 years old (M = 13.52; SD = 1.98). The distribution of participants according to gender, ethnic descent, and region, differentiated by role, is presented in Table 1. The sample size is considered adequate for the analyses performed, allowing stable estimates in multivariate procedures (Hair et al., 2019).

In terms of composition, a differentiated distribution between groups is observed: in the case of teachers, the non-Mapuche population predominates, while in the group of students there is a higher proportion of participants who identify as Mapuche. This configuration is consistent with the intercultural character of the selected establishments.

The selection of the establishments responded to a criterion of intercultural relevance, considering schools with a significant enrollment of Mapuche students (at least 10%), according to records from the Ministry of Education and the National Census (INE, 2025). For the teachers, the inclusion criterion was to work in these establishments. In the case of students, their belonging to courses between 8th grade and 4th grade was considered, and their voluntary participation through informed consent.

### 2.2. Instruments

The Socio-Educational and Cultural Ambivalence Scale (EASC) was used, developed and validated in the context of intercultural education in Chile by Riquelme et al. (2026). This instrument was designed to assess the coexistence of potentially contradictory cultural, cognitive, and affective orientations in the relationship between dominant school knowledge and Mapuche educational knowledge. From a conceptual point of view, the scale is based on the articulation of two complementary theoretical traditions: (a) the model of double educational rationality, which proposes the coexistence of a Western-matrix school rationality and a Mapuche community rationality (Quilaqueo et al., 2016, 2022), and (b) the approach to educational ambivalence as an expression of cognitive, affective, and normative tensions in the face of conflicting value systems (Gasché, 2005; Tabboni, 2008).

The EASC was constructed through a qualitative-quantitative sequential design, which included a conceptual and semantic exploration phase with educational actors (teachers, students, and families), followed by a psychometric validation process. This process made it possible to operationalize the construct of ambivalence as a structural arrangement that reflects tensions between epistemic systems in intercultural school contexts (Riquelme et al., 2026).

The instrument has three conceptually equivalent versions, adapted to the role of the participants in the educational community: EASC-D (teachers); EASC-E (students); EASC-P (Parents/Caregivers).

In the present study, the versions of the scale for teachers and students were used, which maintain conceptual equivalence, with minimal differences in the wording of the items, relevant to the characteristics of each group (Riquelme et al., 2026). It is a five-point Likert-type scale (1 = strongly disagree to 5 = strongly agree).

The structure of the instrument is organized into six dimensions, which capture different expressions of ambivalence in the educational context:

(1) Integration of contextual and cultural knowledge: evaluates the incorporation of Mapuche knowledge into the school curriculum.
(2) School-community linkage and knowledge mobilization: examines the relationship between school, family and community in the transmission of knowledge.
(3) Equal treatment and recognition of cultural diversity: analyzes the valuation of cultural diversity in the school environment.
(4) Cultural identity and resistance to homogenization: evaluates the preservation of cultural identity in the face of pressures of uniformity.
(5) Intercultural education and deepening of traditional knowledge: measures the depth with which Mapuche knowledge is addressed in teaching.
(6) Participation in cultural ceremonies and hybridization spaces: explores participation in cultural practices as intercultural meeting spaces.

Examples of items include: Students: “I like it when we use examples from the Mapuche community to understand the subjects”; Teachers: “I try to include local Mapuche knowledge in the teaching of school content.”

In psychometric terms, the validation of the scale reports adequate confirmatory factor adjustment indices for both the adult and student populations (CFI between 0.93 and 0.98; RMSEA ≈ 0.07), as well as satisfactory levels of internal consistency (ω between 0.70 and 0.97), which supports the structural validity and reliability of the instrument (Chen, 2007; Muthén & Muthén, 2017; Cheung & Rensvold, 2002). Likewise, scalar invariance has been demonstrated between adult groups, which allows comparisons between educational actors (Riquelme et al., 2026).

Overall, the EASC constitutes a valid and reliable instrument for the analysis of the epistemic and cultural tensions that shape educational dynamics in intercultural contexts, allowing its operationalization in comparative studies between school actors.

In this study, the dimensions of the EASC were used as dependent variables to analyze differences in ambivalence according to role and ethnicity.

### 2.3. Procedure

The study was developed in coordination with the participating establishments. The application of the scale was carried out in face-to-face mode, during school days previously authorized by the managements. Each participant received written information about the objectives and procedures of the study, signing consent or informed assent as appropriate, in accordance with the ethical standards of research in education (Sánchez, 1992; Hirsch, 2020). Administration was collective, in groups of 15 to 30 people, under the supervision of a researcher and a trained assistant. The average duration was 25 min. The data were recorded anonymously and encrypted in a secure database, safeguarding the confidentiality and integrity of the participants. The process also considered the return of partial results to the educational communities involved, as part of the ethical dimension.

### 2.4. Analysis Plan

First, mean scores were calculated for each of the six dimensions of the Socio-Educational and Cultural Ambivalence Scale (EASC): (F1) Integration of Contextual and Cultural Knowledge, (F2) School–Community Linkage and Knowledge Mobilization, (F3) Equal Treatment and Recognition of Cultural Diversity, (F4) Cultural Identity and Resistance to Homogenization, (F5) Intercultural Education and Deepening of Traditional Knowledge, and (F6) Participation in Cultural Ceremonies and Hybridization Spaces. These dimensions were treated as continuous dependent variables in the subsequent analyses.

Data distributions were examined through histogram inspection and Q–Q plots, complemented by Kolmogorov–Smirnov and Shapiro–Wilk tests (Ghasemi & Zahediasl, 2012; Razali & Wah, 2011), in order to assess the adequacy of the variables for multivariate analyses.

To address the main objective of the study, a 2 × 2 factorial multivariate analysis of variance (MANOVA) was conducted using educational role (teacher vs. student) and ethnicity (Mapuche vs. non-Mapuche) as fixed factors, and the six dimensions of socio-educational and cultural ambivalence as dependent variables. MANOVA was selected because the dimensions represent theoretically related manifestations of ambivalence and therefore should be examined simultaneously rather than through a series of independent analyses.

The assumption of homogeneity of covariance matrices was evaluated through Box’s M test. Given the significant result obtained, Pillai’s Trace was adopted as the primary multivariate statistic due to its robustness under conditions of covariance heterogeneity (Olson, 1974). Subsequently, factorial univariate analyses of variance (ANOVAs) were performed for each dimension in order to identify the specific variables contributing to the multivariate effects. Effect sizes were reported using partial eta squared (η^2^p).

As a complementary exploratory procedure, a two-step cluster analysis was conducted (Chiu et al., 2001) using the six ambivalence dimensions as clustering variables. This analysis sought to identify recurrent configurations of ambivalence across participants and to examine whether the dimensions formed distinguishable intensity profiles. The optimal number of clusters was determined using the Bayesian Information Criterion (BIC) and the magnitude of change in BIC (ΔBIC), favoring the most parsimonious solution. Cluster quality was assessed through the ratio of distance measures and the silhouette measure of cohesion and separation (Kaufman & Rousseeuw, 1990).

Alternative solutions ranging from two to five clusters were examined during the exploratory phase. The final cluster solution was retained because it provided the best balance between statistical fit, interpretability, and parsimony, as indicated by the mathematical stabilization of the ΔBIC and the highest ratio of distance measures. Solutions with fewer clusters were avoided when they collapsed substantively different levels of ambivalence, whereas solutions with too many clusters were discarded when they produced smaller groups without meaningful theoretical differentiation. In addition, cluster solutions were evaluated based on achieving an acceptable silhouette coefficient (equal to or greater than 0.40) as a benchmark for moderate cohesion and separation among clusters, while ensuring that the retained structure aligned with the objective of identifying theoretically meaningful ambivalence profiles within intercultural educational contexts. The resulting groups were understood as differentiated levels of ambivalence intensity rather than as strictly discrete psychological categories. Finally, the distribution of these profiles according to educational role, ethnicity, and gender was examined through chi-square analyses. All statistical analyses were performed using IBM SPSS Statistics version 27 (IBM Corp., 2020).

## 3. Results

The results are presented in four sequential stages aligned with the objectives of the study. First, descriptive statistics are reported according to educational role and ethnicity. Second, multivariate and univariate analyses are presented to examine differences in socio-educational and cultural ambivalence across groups. Third, simple effects analyses are reported for Mapuche participants in order to compare teachers and students within the same ethnic group. Finally, an exploratory cluster analysis is presented to identify ambivalence profile configurations and their distribution across educational actors.

### 3.1. Descriptive Statistics by Educational Role and Ethnicity

Table 2 presents the descriptive statistics for the six dimensions of socio-educational and cultural ambivalence according to educational role (teachers and students) and ethnicity (Mapuche and non-Mapuche).

Teachers tended to report higher scores than students across most dimensions of ambivalence. Likewise, Mapuche participants generally showed higher scores than non-Mapuche participants. The highest scores were observed among Mapuche teachers, whereas non-Mapuche students consistently reported the lowest levels across the majority of dimensions.

Differences between groups were particularly evident in dimensions associated with the integration of contextual and cultural knowledge (F1), school-community linkage and knowledge mobilization (F2), and participation in cultural ceremonies and hybridization spaces (F6). By contrast, dimensions related to intercultural education and deepening of traditional knowledge (F5) showed smaller differences between Mapuche teachers and students.

These descriptive patterns suggest that both educational role and ethnicity contribute to the distribution of socio-educational and cultural ambivalence, a proposition that was subsequently examined through multivariate analyses.

### 3.2. Multivariate Effects of Educational Role and Ethnicity

To examine whether socio-educational and cultural ambivalence differed according to educational role and ethnicity, a 2 × 2 factorial MANOVA was conducted using role (teacher vs. student) and ethnicity (Mapuche vs. non-Mapuche) as fixed factors, and the six ambivalence dimensions as dependent variables (see Table 3). Given the violation of the homogeneity of covariance matrices assumption (Box’s M = 210.99, *p* < 0.001), Pillai’s Trace was used as the primary multivariate statistic.

The multivariate analysis showed significant main effects of educational role and ethnicity. The effect of role was stronger than that of ethnicity, indicating that teachers and students differed systematically in their overall ambivalence profiles. Ethnicity also showed a significant multivariate effect, suggesting that Mapuche and non-Mapuche participants differed across the combined set of ambivalence dimensions. The role × ethnicity interaction was not statistically significant at the multivariate level, indicating that the effects of role and ethnicity were primarily additive rather than interactive.

### 3.3. Simple Effects Among Mapuche Participants

These results indicate that role and ethnicity independently structure ambivalence, with role showing the largest effect size. Follow-up ANOVAs were conducted for each dimension (see Table 4).

Significant differences were observed in five of six dimensions for Effect of role (F1, F2, F3, F4, F6; all *p* < 0.001), with teachers reporting higher ambivalence than students. No significant effect was found for F5 (*p* = 0.103). Ethnicity significantly affected most dimensions (F1, F2, F4, F5, F6; all *p* < 0.001), with Mapuche participants consistently scoring higher than non-Mapuche participants. F3 did not reach significance. Although the multivariate interaction between role and ethnicity was not significant (see Table 3), univariate analyses revealed small but statistically significant interaction effects in specific dimensions, particularly F4 and F6 (see Table 4). However, effect sizes were small (η^2^p ≈ 0.02), and inspection of descriptive patterns suggests that these differences reflect minor variations in slope rather than substantive changes in the relationship between role and ethnicity. Therefore, interaction effects were interpreted as localized and not central to the overall structure of ambivalence.

Across dimensions, Mapuche participants consistently showed higher mean scores than non-Mapuche participants, a pattern that remained stable after controlling for role. To further examine the role effect within the Indigenous population, simple effects analyses were conducted comparing Mapuche teachers and Mapuche students across the six dimensions of ambivalence (see Table 5).

As shown in Table 5, teachers reported significantly higher scores than students in F1, F2, F3 and F6. No significant differences were observed in F4 and F5. These findings suggest that the higher ambivalence observed among teachers is maintained even when comparisons are restricted to participants sharing the same ethnic background.

### 3.4. Identification of Ambivalence Profiles

A two-step cluster analysis including the six ambivalence dimensions (F1–F6) yielded a three-cluster solution as optimal (BIC = 1425.90; ΔBIC = −301.80; silhouette = 0.40), indicating good cohesion and separation. The clusters were labeled Low, Medium, and High ambivalence. As shown in Table 6, the three profiles differed significantly across all six dimensions (*p* < 0.001), exhibiting a clear monotonic gradient (High > Medium > Low). This pattern supports the existence of structured ambivalence profiles rather than random variation.

### 3.5. Cluster Composition

Cluster membership varied systematically according to educational role, ethnicity, and gender (see Table 7). The High Ambivalence profile included a greater proportion of teachers (38.9%) than students (28.9%), whereas the Low Ambivalence profile was more strongly represented among students (27.5%) than teachers (9.5%). Similarly, Mapuche participants were more frequently concentrated in the High Ambivalence profile (39.4%) than non-Mapuche participants (26.6%), while non-Mapuche participants were more commonly represented in the Medium Ambivalence profile (54.1%). Gender differences were also observed, with women showing a higher proportion in the High Ambivalence profile (37.4%) compared with men (27.8%). Chi-square analyses confirmed significant associations between cluster membership and role, ethnicity, and gender (all *p* < 0.001).

Results indicate that socio-educational and cultural ambivalence is systematically distributed across the educational system. The factorial analyses revealed significant differences associated with both educational role and ethnicity, with teachers generally reporting higher levels of ambivalence than students and Mapuche participants reporting higher levels than their non-Mapuche counterparts. Although the multivariate interaction between role and ethnicity was not statistically significant, specific dimensions showed differentiated patterns that justified further examination through simple effects analyses.

The cluster analysis complemented these findings by identifying three ambivalence profiles that differed primarily in intensity rather than in qualitative structure. The distribution of these profiles across educational actors suggests that ambivalence is not randomly distributed among participants but tends to concentrate according to their educational and sociocultural positions. Taken together, these findings provide an empirical basis for examining socio-educational ambivalence as a structured phenomenon within intercultural educational contexts.

## 4. Discussion

The present study examined socio-educational and cultural ambivalence in intercultural educational contexts, focusing on differences associated with educational role and ethnic belonging. Teachers reported higher levels of ambivalence than students across most dimensions, while Mapuche participants showed significantly higher levels than their non-Mapuche counterparts. Contrary to expectations, the interaction between role and ethnicity was not significant at the multivariate level, suggesting that both factors contribute independently to the distribution of ambivalence. These findings support the interpretation of ambivalence as a structural feature of intercultural schooling rather than as an isolated individual experience.

One of the principal results concerns the effect of educational role, where across most dimensions, teachers reported significantly higher levels of ambivalence than students. This pattern is particularly relevant because the theoretical framework of this study conceptualizes ambivalence as the experiential expression of the tensions generated by the coexistence of different educational rationalities within the same institutional space (Quilaqueo et al., 2017; Quilaqueo & Torres, 2022). From this perspective, teachers occupy a particularly demanding position. Unlike students, whose participation in the educational process is primarily shaped by learning experiences, teachers must constantly mediate between curricular prescriptions, institutional regulations, community expectations, and culturally situated forms of knowledge. Their role therefore exposes them more directly to the contradictions that emerge when different epistemological systems coexist within the school.

The results can be interpreted through the notion of double educational rationality. As proposed by Quilaqueo et al. (2017), intercultural schooling in Mapuche territories is characterized by the coexistence of two partially divergent educational rationalities. On the one hand, the dominant school system privileges standardized, disciplinary, and universal forms of knowledge. On the other hand, Mapuche educational knowledge is organized around relationality, territorial experience, oral transmission, and community participation. Teachers are required to operate at the intersection of these rationalities, often without institutional conditions that allow their effective articulation. Consequently, higher levels of ambivalence among teachers should not be interpreted as indicators of uncertainty or professional weakness, but rather as expressions of their greater exposure to structural epistemic tensions.

The dimensions in which the strongest differences emerged provide further support for this interpretation. The largest effects were observed in the dimensions related to the integration of contextual and cultural knowledge (F1), school–community linkage and knowledge mobilization (F2), and participation in cultural ceremonies and hybridization spaces (F6). These dimensions share a common characteristic: they involve direct engagement with the boundaries between school knowledge and community knowledge. The fact that the strongest differences appear precisely in these dimensions suggests that ambivalence intensifies in contexts where educational actors must actively negotiate the legitimacy, relevance, and applicability of different knowledge systems. In this sense, ambivalence appears to be less associated with abstract cultural identification than with participation in concrete practices of intercultural mediation.

Ethnic belonging also emerged as a significant source of variation where Mapuche participants consistently reported higher levels of ambivalence than non-Mapuche participants across most dimensions. These results are consistent with previous studies suggesting that Indigenous actors frequently navigate multiple normative and epistemological frameworks simultaneously (Quilaqueo et al., 2017, 2022). However, the results also indicate that ambivalence is not exclusively an Indigenous phenomenon. Non-Mapuche participants reported substantial levels of ambivalence across all dimensions, indicating that intercultural educational environments generate tensions that affect the educational community as a whole. What differs is not the existence of ambivalence itself, but the degree to which actors are exposed to the epistemic, cultural, and institutional contradictions that characterize intercultural schooling.

This interpretation is particularly important because it shifts the understanding of ambivalence away from deficit-oriented explanations. Ambivalence should not be viewed as evidence of insufficient cultural adaptation, incomplete intercultural competence, or identity confusion. Rather, it reflects the structural conditions under which educational actors operate. In line with Merton and Barber (1963), Merton (1968), and Tabboni (2008), ambivalence emerges when actors must simultaneously respond to partially incompatible normative expectations. In the present case, these expectations originate from the coexistence of educational systems grounded in different cultural and epistemological assumptions.

Another relevant finding concerns the absence of a significant multivariate interaction between educational role and ethnicity. Although some dimensions showed localized interaction effects, the overall multivariate model indicated that role and ethnicity operated primarily through additive rather than interactive mechanisms. This finding suggests that socio-educational ambivalence is not generated by unique combinations of role and ethnic background. Instead, educational role and ethnic belonging contribute independently to the intensity with which actors experience intercultural tensions. Teachers tend to report greater ambivalence regardless of ethnicity, while Mapuche participants tend to report greater ambivalence regardless of educational role. This pattern points to relatively stable structural dynamics rather than highly specific group configurations.

From a theoretical perspective, this result invites a more nuanced understanding of intercultural educational processes. Much of the literature has tended to assume that intercultural tensions are primarily associated with ethnic differences. The present findings suggest that institutional position may be equally important. The stronger effect associated with educational role indicates that the responsibilities, expectations, and mediating functions assigned to actors within the school system play a crucial role in shaping experiences of ambivalence. Consequently, ambivalence appears not only as a cultural phenomenon but also as an institutional one.

The cluster analysis provides complementary evidence supporting this interpretation. Rather than identifying qualitatively different forms of ambivalence, the analysis revealed three profiles distinguished mainly by their level of intensity. Participants were distributed across low-, medium-, and high-ambivalence profiles, suggesting that ambivalence operates as a continuum rather than as a set of discrete categories. The greater concentration of teachers and Mapuche participants within the high-ambivalence profile reinforces the results obtained through the factorial analyses and indicates that ambivalence is socially patterned rather than randomly distributed.

The convergence between the factorial analyses and the cluster solution strengthens the interpretation of ambivalence as a structured educational phenomenon. While the MANOVA and subsequent ANOVAs identified the contribution of role and ethnicity to the distribution of ambivalence, the cluster analysis revealed how these differences become organized into recognizable profiles of intensity. Together, both approaches suggest that socio-educational ambivalence constitutes a systemic feature of intercultural educational environments, reflecting varying degrees of exposure to the tensions generated by the coexistence of multiple educational rationalities.

If ambivalence reflects the challenges associated with navigating different epistemological frameworks, then educational systems should not seek to eliminate ambivalence but rather create conditions that allow actors to engage with it reflexively and productively. This requires moving beyond additive approaches to interculturality and promoting deeper forms of dialog between school knowledge and Indigenous educational knowledge. Likewise, teacher education programs should provide opportunities to develop competencies related to intercultural mediation, epistemic dialog, and community engagement, particularly in territories characterized by strong Indigenous presence.

### Limitations

Several limitations should be acknowledged. First, the cross-sectional design prevents causal interpretations regarding the development of ambivalence over time. Second, the study was conducted in specific intercultural territories of southern Chile, which limits the generalization of findings to other Indigenous contexts. Third, the analyses relied on self-report measures and therefore reflect participants’ perceptions rather than direct observations of educational practices. Future studies could incorporate longitudinal designs, qualitative approaches, and comparative analyses involving other Indigenous peoples in order to examine how ambivalence evolves across educational trajectories and institutional contexts. Future studies should also incorporate parents and caregivers, whose position between community educational practices and school expectations may provide complementary insights into socio-educational ambivalence.

## 5. Conclusions

The findings suggest that socio-educational and cultural ambivalence constitutes a structural condition of intercultural schooling whose intensity varies according to actors’ institutional and sociocultural positions. Teachers and Mapuche participants do not appear to experience fundamentally different forms of ambivalence, but rather higher levels of exposure to the epistemic and educational tensions generated by the coexistence of multiple educational rationalities. Far from representing a deficit, ambivalence may constitute an inherent condition of intercultural educational life and an indicator of the ongoing negotiations through which educational actors engage with cultural diversity, institutional expectations, and competing forms of knowledge. This perspective contributes to a more complex understanding of intercultural education and highlights the need to recognize ambivalence as a central dimension of educational experience in culturally diverse societies.

## Figures and Tables

**Table 1:** Sample characteristics by role (gender, ethnicity, and region).

Variable	Category	Teachers *n* (%)	Students *n* (%)
Gender	Male	74 (28.2)	136 (47.9)
	Female	188 (71.8)	148 (52.1)
	Total	262 (100)	284 (100)
Ethnicity	Mapuche	80 (30.5)	222 (78.2)
	Non-Mapuche	182 (69.5)	62 (21.8)
	Total	262 (100)	284 (100)
Region	The Araucanía	232 (88.5)	232 (81.7)
	The Lakes	30 (11.5)	52 (18.3)
	Total	262 (100)	284 (100)

Note. Percentages are calculated within each role. Source: Authors.

**Table 2:** Estimated marginal means of socio-educational ambivalence according to educational role and ethnicity.

Dimension	T-M	T-NM	S-M	S-NM
F1. Integration of Contextual and Cultural Knowledge	4.333	4.129	3.883	3.371
F2. School–Community Linkage and Knowledge Mobilization	4.314	4.015	3.801	3.333
F3. Equal Treatment and Recognition of Cultural Diversity	4.603	4.524	4.185	4.011
F4. Cultural Identity and Resistance to Homogenization	4.244	3.926	4.071	3.417
F5. Intercultural Education and Deepening of Traditional Knowledge	4.184	3.758	4.199	3.500
F6. Participation in Cultural Ceremonies and Hybridization Spaces	4.336	4.101	4.025	3.372

Note. T = Teacher; S = Student; M = Mapuche, NM = Non-Mapuche.

**Table 3:** Multivariate Effects of Role and Ethnicity on Socio-Educational and Cultural Ambivalence (MANOVA).

Effect	Pillai’s Trace	F (df1, df2)	*p*	η^2^p
Role	0.230	26.67 (6, 537)	<0.001	0.230
Ethnicity	0.108	10.86 (6, 537)	<0.001	0.108
Role × Ethnicity	0.019	1.77 (6, 537)	0.104	0.019

Source: Authors.

**Table 4:** Univariate Effects of Role and Ethnicity on Ambivalence Dimensions.

Dimension	Role F	*p*	η^2^p	Ethnicity F	*p*	η^2^p	Interaction F	*p*	η^2^p
F1	75.41	<0.001	0.122	26.48	<0.001	0.047	4.89	0.027	0.009
F2	74.37	<0.001	0.121	30.58	<0.001	0.053	1.47	0.226	0.003
F3	53.09	<0.001	0.089	3.89	0.049	0.007	0.55	0.459	0.001
F4	18.49	<0.001	0.033	37.70	<0.001	0.065	4.52	0.034	0.008
F5	2.67	0.103	0.005	57.49	<0.001	0.096	3.41	0.066	0.006
F6	46.50	<0.001	0.079	33.93	<0.001	0.059	7.53	0.006	0.014

Source: Authors.

**Table 5:** Simple Effects Analysis: Comparison Between Mapuche Teachers and Mapuche Students Across Ambivalence Dimensions.

Dimension	Teacher M	Student M	ΔM	*p*	Cohen’s d
F1	4.33	3.88	0.45	<0.001	0.63
F2	4.31	3.80	0.51	<0.001	0.72
F3	4.60	4.18	0.42	<0.001	0.63
F4	4.24	4.07	0.17	0.105	0.21
F5	4.18	4.20	−0.02	0.870	−0.02
F6	4.34	4.03	0.31	0.003	0.39

Source: Authors.

**Table 6:** Differences Between Ambivalence Profiles Across Dimensions.

Dimension	Low M (SD)	Medium M (SD)	High M (SD)	F (2, 543)	*p*
F1	2.96 (0.67)	3.90 (0.42)	4.64 (0.37)	443.20	<0.001
F2	2.90 (0.59)	3.81 (0.42)	4.58 (0.41)	460.08	<0.001
F3	3.48 (0.66)	4.37 (0.48)	4.78 (0.42)	220.42	<0.001
F4	2.97 (0.83)	3.87 (0.49)	4.69 (0.45)	322.87	<0.001
F5	3.10 (0.83)	3.82 (0.52)	4.67 (0.38)	278.08	<0.001
F6	2.91 (0.78)	3.96 (0.43)	4.74 (0.33)	468.08	<0.001

Note. Bonferroni post hoc tests confirmed significant differences between all groups. Source: Own elaboration.

**Table 7:** Distribution of Ambivalence Profiles by Role, Ethnicity, and Gender.

Variable	Category	Low (%)	Medium (%)	High (%)	*p*-Value (χ^2^)
Role	Teacher	9.5	51.5	38.9	<0.001
	Student	27.5	43.7	28.9	
Ethnicity	Mapuche	18.5	42.1	39.4	<0.001
	Non-Mapuche	19.3	54.1	26.6	
Gender	Male	28.3	43.9	27.8	<0.001
	Female	12.9	49.7	37.4	

Source: Authors.

## Data Availability

Data are not available due to ethical restrictions.
